# Novel Insights into the Wattle and Daub Model of *Entamoeba* Cyst Wall Formation and the Importance of Actin Cytoskeleton

**DOI:** 10.3390/pathogens13010020

**Published:** 2023-12-24

**Authors:** Deepak Krishnan, Meenakshi Pandey, Santoshi Nayak, Sudip K. Ghosh

**Affiliations:** Department of Bioscience and Biotechnology, Indian Institute of Technology Kharagpur, Kharagpur 721302, West Bengal, India; deepak.lex@gmail.com (D.K.); meenakshi901@gmail.com (M.P.); santoshinayak10@gmail.com (S.N.)

**Keywords:** encystation, *Entamoeba invadens*, chitin synthase, cortical actin, cyst wall, chitin, lectin

## Abstract

The “Wattle and Daub” model of cyst wall formation in *Entamoeba invadens* has been used to explain encystment in *Entamoeba histolytica*, the causal agent of amoebiasis, and this process could be a potential target for new antiamoebic drugs. In this study, we studied the morphological stages of chitin wall formation in *E. invadens* in more detail using fluorescent chitin-binding dyes and the immunolocalization of cyst wall proteins. It was found that chitin deposition was mainly initiated on the cell surface at a specific point or at different points at the same time. The cystic wall grew outward and gradually covered the entire surface of the cyst over time, following the model of Wattle and Daub. The onset of chitin deposition was guided by the localization of chitin synthase 1 to the plasma membrane, occurring on the basis of the Jacob lectin in the cell membrane. During encystation, F-actin was reorganized into the cortical region within the early stages of encystation and remained intact until the completion of the chitin wall. The disruption of actin polymerization in the cortical region inhibited proper wall formation, producing wall-less cysts or cysts with defective chitin walls, indicating the importance of the cortical actin cytoskeleton for proper cyst wall formation.

## 1. Introduction

The protozoan *Entamoeba histolytica* is the causative agent of amoebiasis, the third most important parasitic disease after malaria and schistosomiasis, and causes 40–50 million cases and 40,000–100,000 deaths annually [1]. Nitroimidazole-derivative drugs such as metronidazole, tinidazole, and ornidazole are the drugs of choice for the treatment of amoebiasis with serious side effects. Although no cases of amoebiasis being resistant to these drugs have been reported, it was possible to generate strains of *E. histolytica* resistant to these drugs in vitro [2,3], indicating that the widespread use of anti-amoebic drugs may eventually cause drug resistance. Thus, it is necessary to find new, safer drugs, and, for this purpose, novel drug targets are required to be identified. *Entamoeba histolytica* reaches new hosts through the ingestion of water or food contaminated with cysts. Thus, developing a chemotherapeutic agent that blocks encystation can prevent the spread of the parasite by reducing the number of cysts in the environment. The events of cyst wall deposition are of particular interest in this regard, as it is the wall that provides a resistant nature to the cyst. Immunization with cyst wall proteins of *Giardia lamblia* was reported to cause a significant reduction in cyst shedding in murine models [4,5]. To find such drug or vaccine candidates, it is necessary to understand the structure of the chitin wall and the mechanics of its formation.

Since *E. histolytica* does not encyst in an axenic culture, though a recent study reported the occurrence of encystation in *E. histolytica* [6], the reptilian parasite *Entamoeba invadens* was opted for as a model for studying encystation, as it readily forms cysts when subjected to starvation and osmotic stress [7]. *E. invadens* has a similar genome, occupies a similar host niche, and causes a colon pathology similar to that of *E. histolytica* [8,9]. Most importantly, the cyst characteristics of *E. histolytica* and *E. invadens* are quite similar: both formed a chitin-walled tetranucleate cyst with a single chromatoid body [10]. A comparison of gene expression during encystation showed that they share a large number of developmentally regulated genes. Encystation-specific genes of *E. invadens* were also found to be overexpressed in clinical isolates of *E. histolytica* [11]. Immunolocalization experiments showed that the cyst wall proteins identified in *E. invadens* such as chitinase, the Jacob lectin, and the Jessie lectin are also present in *E. histolytica* cysts isolated from patients [12].

Unlike the fungal wall that contains multiple layers of sugar polymers, including alpha-glucans, beta-glucans, and chitin [13], the *Entamoeba* cyst wall contains just one layer composed of sugar polymer chitin, which constitutes 25% of its total dry weight, and other proteins such as chitinase and Jacob and Jessie lectins [14,15]. Chitin is a homopolymer of a β-(1,4)-linked N-acetyl-D-glucosamine. In the cyst wall, it is associated with the chitin-binding domains (CBDs) of three encystation-specific glycoproteins. Of the three proteins, Jacob and Jessie lectins contain 6-Cys and 8-Cys CBDs, respectively [16,17]. The third protein is a chitinase enzyme that modifies chitin and contains 8-Cys CBDs. The genome of *E. invadens* encodes seven Jacob lectins (Jacob1–7) with three–five CBDs, two Jessie lectins (Jessie 3a and 3b), and one chitinase (Chitinase 1) featuring a single CBD at the N-terminal. The inclusion of chitin-binding domains (CBDs) in lectins implies their involvement in linking lectins to the chitin fibril [18].

The “Wattle and Daub model” was previously used to explain the assembly of different components, including chitin fibrils and lectins, into the cyst wall [19]. According to this model, *Entamoeba* cyst wall synthesis takes place in three phases. First, during the foundation stage, the Jacob lectin is secreted and binds to constitutively expressed Gal/GalNAc lectins on the cell membrane through its Galactose (Gal) residue. Next, during the “wattle” stage, chitin synthases produce chitin fibrils using UDP-GlcNAc provided by nucleotide sugar transporters [20]. These chitin fibrils are then cross-linked by the Jacob lectin through their chitin-binding domains (CBD), forming the framework of the wall. However, Jacob lectins and chitin are synthesized early during encystation but in distinct vesicles [19]. Chitinases trim the tangentially protruding chitin fibrils by degrading the β-(1,4) linkage between the N-acetylglucosamine residues of chitin, and chitin deacetylase deacetylates the chitin fibrils to form chitosan, which is resistant to hydrolysis by chitinases [21]. Finally, during the daub phase, the sticky Jessie lectin, which has a single CBD and a self-aggregation-inducing C-terminal domain, binds to this framework, completing the cyst wall and making it impermeable [19].

According to the “Wattle and Daub” model, the addition of Jessie lectins occurs after 36 h of encystation in the “daub stage”. However, the expression profiles of cyst wall proteins showed that most of them were overexpressed between 8 and 24 h [22,23]. In order to understand how different components were added to the wall, the intermediate stages of cyst wall formation were investigated. As in vitro encystation is an asynchronous process, it is difficult to follow its progression. So we studied the deposition of chitin by staining cells with chitin-binding dyes and analyzed chitin’s maturity based on detergent resistance. Localizing the chitin allowed us to follow the highly asynchronous encystation, as chitin is the most important marker of encystation. We also studied cyst wall deposition, with respect to the appearance of other cyst characteristics like chromatoid body formation and nuclear division.

The relationship between the actin cytoskeleton and chitin wall formation is well-established in fungi. Actin was found at sites where cell wall synthesis is active, like the bud, growing apices, and sites of septum formation [24]. In regenerating protoplasts of *Saccharomyces cerevisiae*, actin patches were found over the surface on which a new cell wall was being synthesized [25]. During encystation, *E. invadens* undergo morphological changes from highly motile trophozoites to an immobile spherical cyst, which shows the involvement of the cytoskeleton during stage conversion. In the case of *Entamoeba*, morphology and motility are entirely controlled by the actin cytoskeleton, as cytoplasmic microtubules are absent [26]. Also, actin inhibitors were shown to block *E. invadens* encystation [27,28]. So the role of actin in *Entamoeba* cyst chitin wall deposition was also investigated.

## 2. Materials and Methods

### 2.1. Cells and Reagents

*Entamoeba invadens* strain IP-1 was maintained in TYI-S-33 medium containing 10% adult bovine serum (HiMedia, Mumbai, India), 3% Diamond vitamin mix, and 1% streptomycin–penicillin G (100×, Himedia, India) at 25 °C [29]. DAPI, propidium iodide (PI), calcofluor white (CFW), Alexafluor 633-tagged wheat germ agglutinin (WGA), acridine orange, and cytochalasin D were purchased from Sigma-Aldrich (St. Louis, Missouri, United States). 2,3-Butanedione monoxime (BDM) was purchased from HiMedia, India. TRITC-conjugated phalloidin was purchased from Molecular Probes, Invitrogen, Waltham, MA, USA. Antibodies against chitinase (EIN_096870) were raised in rabbits in our own animal house facility [30], whereas chitin synthase 1 (EIN_066020) antibodies were commercially raised in rabbits by ABGENEX Pvt. Ltd. in India. Jacob (EIN_230100) and Jessie (EIN_066080) antibodies were kindly provided by John Samuelson.

### 2.2. Antibody Production against E. invadens

#### Chitin Synthase 1

In order to generate specific antibodies for the localization of chitin synthase 1 (EiCHS1), a gene fragment encoding a part of the catalytic domain in EiCHS1 was cloned and heterologously expressed in bacterial cells as a protein of 17.5kD. Primers 5′GGATCCCACTCGCACGAAATCTTCTTT3′ and 5′CTCGAGGTACCCTGTATCCCACCCAG 3′ were used to amplify a region from 921bp to 1374bp of Eichs1 gene, and the amplified product was cloned into pET-21a (+) vector. The construct was transformed into C43 (DE3) bacterial strains for protein expression. The polyclonal antibody against this expressed protein was commercially raised in rabbits by ABGENEX Pvt. Ltd., Bhubaneswar, India.

### 2.3. Plasmid Construction and Transfection

A trigger-mediated RNA-interference gene silencing approach was used to downregulate EiHbox1 (EIN_475530), as previously published by Meenakshi et al., 2023 [31]. In brief, the 152-trigger region was inserted into the pEi-CKII-Luc plasmid (a kind gift from Upinder Singh) using the *Nhe*I restriction site. To create the 5′ trigger construct, the entire sequence of the EiHbox1 gene was added downstream of the 152-trigger region, utilizing the *Avr*II and *Sac*II restriction sites. Subsequently, *E. invadens* trophozoites were transfected with this silencing construct through electroporation. The stable transfected cells were then maintained in the presence of G418 at a concentration of 20 μg per mL.

### 2.4. Encystation

To prepare the encystation (47% low glucose or LG 47) medium, TYI-S-33 medium without glucose was prepared, diluted 2.12 times, and then completed with 5% heat-inactivated adult bovine serum, 1.5% Diamond vitamin mix, and 1% streptomycin–penicillin G antibiotic (100×, HiMedia, Mumbai, India). Mid-log phase trophozoites were chilled on ice for 10 min to detach the cells from the culture tube wall and harvested by centrifugation at 500× *g* for 5 min at 4 °C. These cells were washed 3 times with LG 47, and then the cell number was adjusted to 5 × 10^5^ trophozoites per ml. Finally, 2 mL of culture was added to each well of a 24-well plate, sealed with parafilm tape, and incubated at 25 °C for different number of hours [32].

### 2.5. Estimation of Encystation Efficiency

Encystation efficiency was calculated in two different ways, one based on detergent resistance and the other based on the number of chitin-positive cells after staining with fluorescent chitin-binding dyes. To estimate the encystation efficiency from detergent resistance, cells were harvested from the encystation culture and were counted using a hemocytometer (Marienfeld, Lauda-Konigshofen, Germany). They were treated with 0.1% Sarkosyl for 10 min on ice to remove trophozoites, and then the number of detergent-resistant cysts was counted, from which the encystation efficiency was calculated by dividing the number of cysts formed by the total number of cells (trophozoites plus cysts) utilized for encystation. To measure the percentage of cysts with incomplete and complete chitin walls, calcofluor white (CFW)-stained (30 µM) encystation culture was taken on a slide, and images were taken at random under phase contrast and UV light. From these images, the percentage of fluorescing cysts, both incomplete and complete, was calculated. To observe the formation of cysts inside the aggregates, 30 µM CFW was directly added to the encystation medium, incubated for 10 min, and then observed under the fluorescence microscope. Each encystation experiment was repeated a minimum of three times, and at least 60 cells were counted to calculate encystation efficiency at each time point.

### 2.6. Cell Staining

To stain the chitin wall, the cells were treated with CFW at 30 µM concentration for 5 min at room temperature (RT) without shaking before fixing the cells, as the dead cells were observed to non-specifically stain. The chitin wall was also stained with Alexafluor 633-conjugated wheat germ agglutinin (WGA) at a concentration of 20 µg per ml. After PBS wash to remove the CFW or WGA, cells were fixed with 4% (*w*/*v*) paraformaldehyde in PBS for 10 min and then permeabilized in 0.5% (*v*/*v*) Triton X-100 in PBS for 5 min at RT. DAPI (0.5 µg/mL), Sytox Green (1 µM), and PI (10 µg/mL) were used to stain the nucleus. Acridine Orange at 10 µM concentration and fluorescein isothiocyanate (by non-specific binding) were used to stain the chromatoid body. For actin localization, permeabilized cells were blocked with 2% (*w*/*v*) BSA for 30 min at RT and stained with TRITC-conjugated phalloidin (1:300) (Molecular Probes, Invitrogen, USA) for 1 h at RT. To localize the cyst wall proteins, CFW-stained, fixed, and permeabilized cells were blocked with 2% (*w*/*v*) BSA and then incubated with appropriate dilution (1:300 for Jacob antibody and 1:100 for all other antibodies) of cell wall protein antibody at 4 °C overnight followed by fluorescein isothiocyanate (FITC)-conjugated goat anti-rabbit secondary antibody (1:500) for 1 h at RT and examined by fluorescence and confocal microscopy.

### 2.7. Microscopy

Olympus IX51 inverted light microscope with camera attachment and photo-editing software (Image Pro Discovery; version 10) and Olympus FV1000 and FV3000 confocal microscopes with Fluoview software (versions 4.2 and 2.6, respectively) were used for fluorescence imaging. Image analysis was then completed using ImageJ software; version 1.51t (NIH). To find the co-localization of chitin fibrils with cyst wall lectins and chitin synthase, the RGB Profiler plugin was used.

### 2.8. RNA Isolation and RT-PCR

Total RNA was isolated after different numbers of hours of encystation culture using TRIzol reagent (Ambion Inc., Austin, TX, USA). After treating total RNA with DNaseI for 60 min, followed by inactivation for 10 min at 80 °C (Takara Bio Inc., Kusatsu, Japan), first-strand cDNA was synthesized from 3 µg of DNaseI-treated RNA by using MuLV super RT reverse transcriptase (BioBharati Lifescience, Kolkata, India), according to the manufacturer’s specifications. The cDNA synthesis was carried out for 50 min at 42 °C, followed by the inactivation of the enzyme for 15 min at 70 °C. Using first-strand cDNA as a template, semi-quantitative RT-PCR was completed to find the expression of chitin wall proteins using Premix Taq (Xcelris, Ahmedabad, India) DNA polymerase and the following primer pairs mentioned in Table 1. The housekeeping gene *E. invadens* ADP Ribosylation Factor (EiARF) was chosen as a reference gene in RT-PCR because it maintains a consistent level of expression across both vegetative and encystation stages [31]. The PCR products were analyzed by running in a 1.5% agarose gel.

## 3. Results and Discussion

### 3.1. Chitin Wall Is Assembled on the Surface of Encysting Cells Starting from Distinct Point(s)

**Figure 1:**
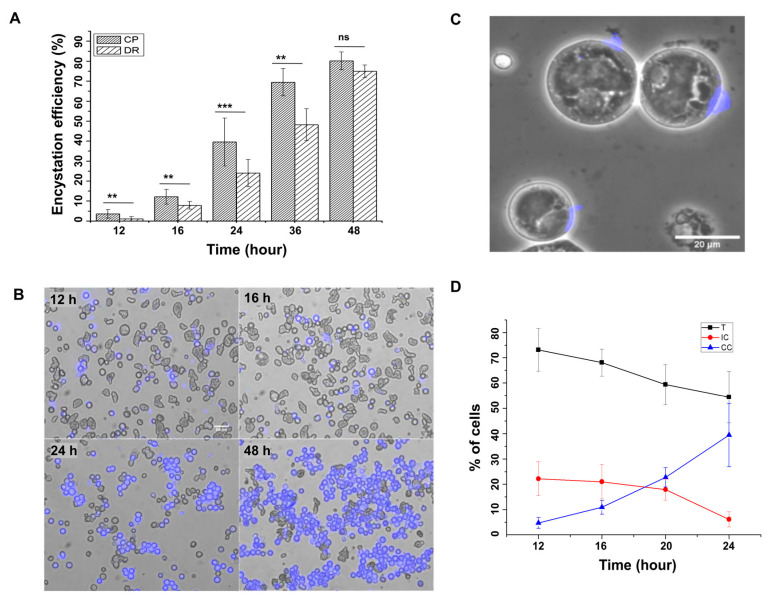
Cysts with incomplete chitin walls represent the intermediate stages in chitin wall formation. (**A**) During the early hours of encystation, the number of detergent-resistant (DR) cells was less than the number of chitin-positive (CP) cells, even though both depended on the presence of chitin. (**B**) Encystation culture after 12, 16, 24, and 48 h of being treated with calcofluor white (CFW, Blue) shows cells with partial chitin wall in the early hours. Scale bar: 50 µm. (**C**) These cells with partial chitin walls could be the reason for the difference in the number of detergent-resistant cells and chitin-positive cells. Scale bar: 20 µm. (**D**) Percentage of trophozoites (T), cysts with incomplete walls (IC), and complete walls (CC) after 12, 16, 20 and 24 h. Blue color represents chitin. (Data are shown as mean ± SD for a minimum of 3 independent experiments. ** *p* < 0.01, *** *p* < 0.001; ns = not significant).

The in vitro encystation of *E. invadens* is an asynchronous process and takes nearly 48–72 h to form a mature tetranucleated cyst. The progression of encystation is usually estimated by calculating the percentage of detergent-resistant cysts. Cysts become detergent-resistant only after the completion of the cyst wall. The early wall-forming stages were not resistant to detergent, as, after staining with calcofluor white (CFW), the chitin-binding dye showed that the numbers of detergent-resistant cells were less than the total number of CFW-positive encysting cells, especially in the early hours of encystation (Figure 1A). In order to identify the chitin-positive but detergent-sensitive cells, the encystation culture was examined at different time points (0 to 48 h) by staining the chitin wall with CFW (Figure 1B). No fluorescence was observed on the encysting cells until the 9th hour (figure not shown), but many cells with intense CFW fluorescence spots or cells with fluorescence covering only a portion of their surface were observed between the 12 and 16th hour’s encystation culture, indicating the initiation of cyst wall formation. By 24–48 h, fluorescence was found all over the cell surface. Cells with incomplete chitin walls (Figure 1C) could be the intermediates in cyst wall formation. The numbers of such cells were manually counted using microscopy. The percentages of trophozoites (T), intermediate cysts with a partial wall (IC), and cells with a complete or nearly complete wall (CC) are given in Figure 1D. After up to 20 h of encystation, intermediate cysts (IC) comprised nearly 20% of the total cells and gradually decreased to about 5% at the 24th hour. These observations confirm that chitin deposition started after 9 h and actively took place between 12 and 24 h of encystation. All the chitin biosynthesis pathway genes and the genes of all the other known protein components of the cyst wall were also found to be overexpressed during the same interval of encystation [22,23].

**Figure 2:**
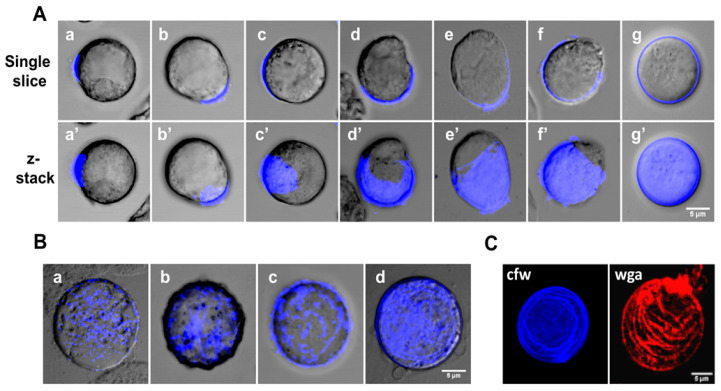
Possible sequence of chitin wall deposition. (**A**) All possible intermediates of chitin wall formation obtained from 9–16 h of encystation were observed with confocal microscopy. The upper panel (a–g) shows a single slice, and the lower panel (a’–g’) shows a z-stack. (**B**) In a few cells (1–2%), chitin formation started at multiple points (a), and their size increased (b,c), finally forming linear arrays on the cell surface (d). (**C**) CFW (Blue) and WGA (Red) staining both showed that chitin is deposited on the cell surface in a patterned manner. Blue and red colors represent the chitin. Scale bar: 5 µm.

The comprehensive observation and analysis of CFW-stained early encystation culture with confocal microscopy helped us to find a good number of possible intermediates of chitin wall formation. A probable sequence of wall formation is shown in Figure 2A. In most cells, wall formation was observed to start at one point, from which the chitin fibrils grew outwards and covered the whole surface. Similar cell wall formation was also observed in regenerating protoplasts of tobacco [33] and *Schizosaccharomyces pombe* [34,35]. This pattern of chitin deposition might be due to the movement of wall-synthesizing machinery at the site of wall formation, resembling the movement of the cellulose synthase complex in plants [36], or may be due to the self-assembly of wall components from a single point. Also, in a few encysting cells (1–2%), the chitin wall seemed to start from multiple points and then spread all over the surface, finally overlapping and forming a continuous cyst wall (Figure 2B). Also, staining with CFW and wheat germ agglutinin (WGA) showed that chitin is deposited on the cyst surface in a patterned manner (Figure 2C).

**Figure 3:**
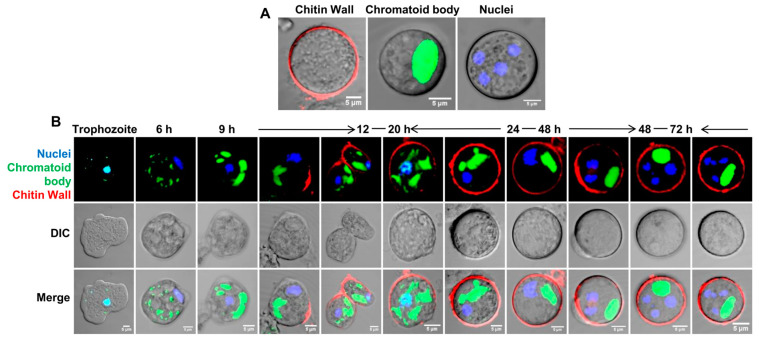
Chitin wall deposition with respect to nuclear division and chromatoid body formation. (**A**) The features of a mature *Entamoeba* cyst, chromatoid body, nucleus, and chitin wall, shown by staining with acridine orange (green), DAPI (blue), and WGA (red), respectively. (**B**) Simultaneous observation of the chromatoid body, nucleus, and chitin wall in encysting cells showed that the chromatoid body appeared by the 6th hour as small aggregates, which then coalesced with each other with time. Chitin wall deposition occurred between 12 to 20 h, and, by the completion of the chitin wall, the chromatoid body was also fully formed. The chitin wall and chromatoid body were completed within 24–48 h. Nuclear division after the formation of the chitin wall and chromatoid body produces the mature tetranucleated cyst. Blue, green, and red colors represent the nucleus, chromatoid body, and chitin, respectively. Scale bar (white line): 5 µm.

Apart from the chitin wall, the other important characteristics of a mature cyst are the formation of four nuclei and the chromatoid body (Figure 3A). To find out how these characteristics appear in cysts with respect to wall formation, the chitin wall, chromatoid body, and nucleus were simultaneously stained (Figure 3B). The chromatoid body and the nuclei were specifically stained with acridine orange and DAPI, respectively, and Alexafluor 633-tagged wheat germ agglutinin was used to stain the chitin wall. The chromatoid body was the first feature to appear in encysting cells; it appeared as multiple small green fluorescing structures in the cytoplasm after 6 h that, with time, coalesced with each other to form a single compact structure. Chitin wall deposition took place between 12 and 20 h and was completed by the 24th hour, during which the chromatoid body acquired its final form. By the 48th hour, the nuclear divisions were completed to produce the mature tetranucleated cyst.

### 3.2. Localization of the Chitin Synthase on the Cyst Surface Guides Chitin Deposition

**Figure 4:**
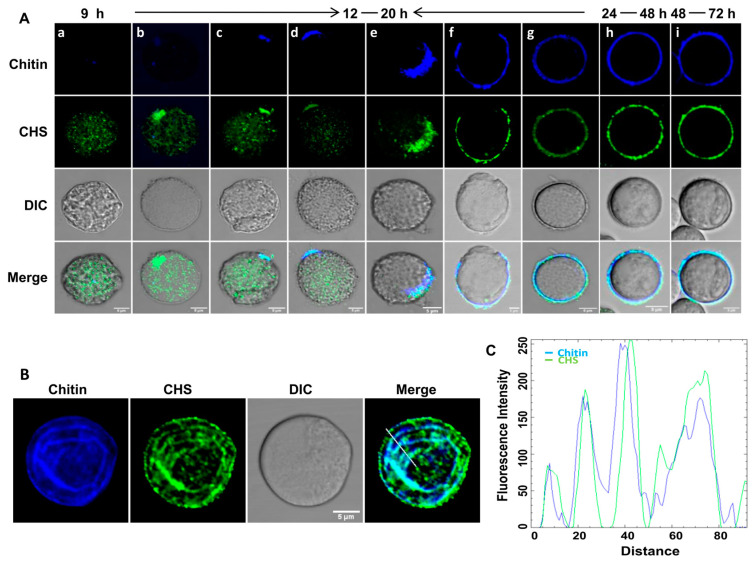
EiCHS1 determines the site of chitin deposition. Immunolocalization of chitin synthase during encystations. (**A**) Chitin synthase was observed in a small vesicle (punctuate staining) in the cytoplasm in the 9th hour of encysting cells (a). It was observed to accumulate at a certain spot of the cell membrane, and chitin formation was initiated at that point. This incomplete chitin wall then grew around the cell along with the chitin synthase. Once the wall was completed, chitin synthase co-localized with chitin (b–g). In 24th-hour cysts with complete walls and 48th-hour mature cysts, it remained embedded in the wall (h,i). (**B**) On the cyst surface, chitin staining by CFW (blue) showed the presence of linear arrays of chitin fibrils that co-localized with EiCHS1 (green). (**C**) Co-localization of EiCHS1 with chitin matched these patterns, which can be seen from the plot of intensity values along the line drawn across the image. Blue and green colors represent chitin and chitin synthase 1, respectively. Scale bar (white line): 5 µm.

*Entamoeba invadens* contains two chitin synthases with different properties, and expression of both chitin synthase genes increased during in vitro encystation [37]. In the present work, we cloned and expressed a truncated chitin synthase 1 (EiCHS1) to raise polyclonal antibodies in rabbits, which were then used for immunofluorescence microscopy (Figure 4A). EiCHS1 was not observed in the trophozoite stage, but by the 9th hour EiCHS1-loaded small vesicles were localized in the cell, distributed throughout the cytoplasm (Figure 4A—a). As the encystation progressed, by 12–16 h, EiCHS1 was found to be concentrated at one site (Figure 4A—b), which was found to be the starting point for chitin deposition (Figure 4A—c). Chitin synthases isolated from the tobacco hornworm’s midgut and mollusks were found to form a high-molecular-mass oligomeric complex [38,39]. The cellulose synthase of plants formed a “rosette” complex to synthesize cellulose microfibrils [36]. In *Entamoeba*, similar interactions between multiple chitin synthases on the membrane may be required for the secretion of chitin microfibrils. With time, more and more chitin synthase reached the plasma membrane and added chitin to the pre-existing chitin wall, extending it and finally covering the entire surface (Figure 4A—d–g). In cysts with complete walls obtained after 24 h of encystation (Figure 4A—h) and in mature cysts after 48–72 h (Figure 4A—i), EiCHS1 co-localized with the chitin wall. The surface of the *Entamoeba* cyst usually contained an array of linear chitin fibrils, which can be seen from CFW staining (Figure 4B). This patterned chitin deposition on the cyst surface was also reflected in the immunolocalization of chitin synthase (Figure 4B,C). These observations show that chitin wall deposition may be guided by chitin synthase localization. EiCHS1 synthesized the chitin fibrils at the cell surface, and, once the wall was completed, it remained associated with the chitin wall. Such localization of the chitin synthases in the wall itself was also reported in fungi like *Mucor rouxii* and *Ustilago maydis* [40,41]. Whether chitin synthases are secreted to the wall or remain physically trapped in the secreted chitin chains [42] is not clear. Thus, like the other lectin components, chitin synthase also becomes part of the wall instead of remaining in the plasma membrane.

Several authors showed that during encystation, chitin was made in intracellular secretory vesicles and then deposited on the cyst wall [19,43]. In this study, we demonstrated that in the cyst, chitin is also synthesized by a membrane-bound chitin synthase enzyme. Recently, a very similar model for cellulose microfibril synthesis by membrane-bound cellulose synthase was proposed in *Acanthamoeba* cyst [44]. Individual chitin synthase enzymes synthesize microfibrils of differing structures at specific locations in the *Candida albicans* cell wall [45]. We believe both processes are simultaneously taking place in *Entamoeba*. To obtain the complete picture of chitin synthesis and deposition, the role of chitin synthase 2 also needs to be explored.

### 3.3. Revisiting the Wattle and Daub Model of the Cyst Wall (Chitin, Chitinase, and Jessie Lectin Assemble into the Cyst Wall from One Starting Point on the Foundation Made of Jacob Lectin)

**Figure 5:**
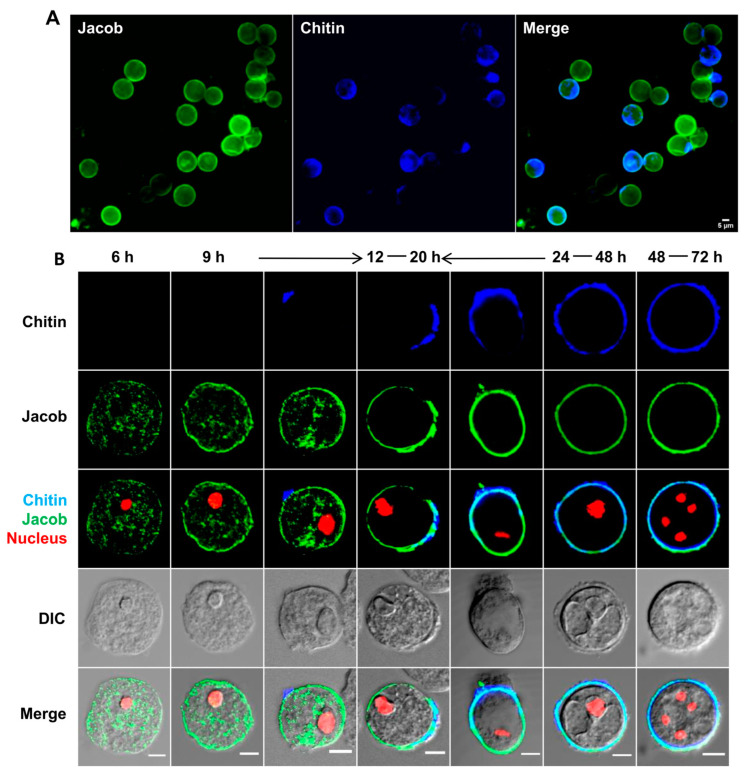
Immunolocalization of Jacob during encystation. (**A**) Twelve hour encystation culture showing the presence of Jacob lectin (green) all over the surface of most cells, with chitin being deposited on the cell surface starting from one point. (**B**) Jacob lectin was observed in the vesicles in the cytoplasm by the 6th hour, and, by the 9th hour, it was found on the membrane. Between 12 and 20 h, chitin wall was deposited on this Jacob layer, starting from one point. In the 24th-hour and 48th-hour mature cysts, it co-localized with the chitin cyst wall as it became a part of the wall. Chitin and nucleus were stained with CFW (blue color) and PI (red color), respectively. The blue, green, and red colors represent chitin, Jacob lectin, and nucleus, respectively. Scale bar (white line): 5 µm.

**Figure 6:**
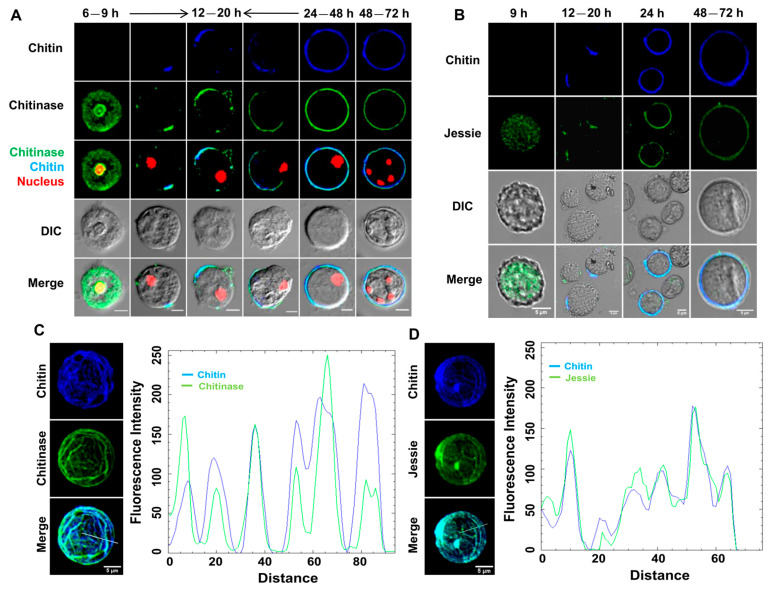
Immunolocalization of chitinase and Jessie during encystation. (**A**) Chitinase lectin (green) was observed in the cytoplasm by the 6th hour. In cysts with incomplete walls obtained from 12 to 20 h, chitinase always co-localized with the chitin. After the wall was completed, it remained embedded in the wall. (**B**) Jessie lectin (green) was also found in the incomplete wall after 12–20 h in encystation culture. It was also found in cysts with complete walls after the 24th hour and in mature cysts after 48–72 h of encystation. (**C**,**D**) Chitin was deposited in a patterned manner on the cyst surface, as observed by CFW staining, and this pattern was also reflected in chitinase (**C**) and Jessie (**D**). The plot of intensity values along the line drawn across the confocal images shows the co-localization of chitin with lectins, indicating their localization is determined by chitin deposition, as these lectins bind to chitin fibrils through their chitin-binding domains. Chitin and nucleus were stained with CFW (blue color) and PI (red color), respectively. The green, blue, and red colors represent the cyst wall proteins, chitin, and nucleus, respectively. Scale bar (white line): 5 µm.

In this study, we revisited cyst wall formation and noticed the same sequence of events during cyst wall formation as referred to in the Wattle and Daub model [19]. Here, we followed encystation by co-localizing known cyst wall proteins like Jacob, Jessie, and chitinase, using their respective antibodies [19,30,46] with the growing chitin wall. Co-localizations of Jacob, chitinase, and Jessie, independently with chitin at different time points of encystations, are demonstrated in Figure 5 and Figure 6A,B, respectively. This study indicates that the composition of the complete, as well as the incomplete wall, was in accordance with the Wattle and Daub model with respect to cyst wall composition, the sequences of events, and the organization of the components.

Contrary to the previously described model, we noticed that Jessie expresses in about the 9th hour of encystation and is also found to co-localize with the growing chitin wall (Figure 6B). The early expression of Jessie is also in consensus with the expression profile of different cyst wall proteins, including Jessie [22,23]. However, the expression of Jessie is relatively low and hard to detect unless it is co-localized with chitin. We believe that in all the previous reports of cyst wall biosynthesis, partial cyst wall formation was overlooked and so was the expression of Jessie during the early hours of encystations. This is the first report where all cyst wall proteins (CWP) were co-localized with cyst wall chitin, and that helped us to clearly spot the CWP.

Our observations clearly indicate that the Jacob and chitin synthase deposition on the cell surface is chitin-independent, whereas the deposition of chitinase or Jessie is chitin-dependent. Encysting cells with ridge-like chitin all over the surface, along with co-localized chitinase and Jessie, were also visible (Figure 6C,D). These woolen-ball-like structures were also reported by Chatterjee et al., 2009 [19]. These cells are most likely originated by the multipoint initiation of cyst wall formation and are also much brighter with respect to cells with partially formed walls under the fluorescence microscope. The population of these brighter cells is much more attractive to be imaged, so less-bright populations might be ignored in previous reports.

Encystation only took place inside the multicellular aggregates found in the encystation culture [32], and it could be possible that the components of the cyst wall were secreted into the intercellular space where they underwent self-assembly into a complete chitin wall on the cell surface from a starting point. The Jessie lectin has a unique C-terminal domain that appears to promote self-aggregation [19]. Also, the ionic attraction between negatively charged Jacob, Jessie, and chitinase and positively charged chitin/chitosan could be mediating such self-assembly. This may also explain the importance of cell aggregation in cyst formation, as, within the aggregate, the concentration of these secreted cell wall components would be high, promoting cell wall assembly [47].

### 3.4. During Encystation, F-Actin Was Reorganized into the Cortical Region Where It Remained until the Completion of the Chitin Wall

In the trophozoite stage of *Entamoeba*, the dynamic cytoskeleton is involved in motility, pseudopodia formation, and phagocytosis. During encystation, *E. invadens* undergoes morphological changes from highly motile trophozoites to an immobile spherical cyst, which shows the involvement of the actin cytoskeleton in stage conversion. *Entamoeba* lacks cytoplasmic microtubules, so the cell morphology is determined by the actin cytoskeleton alone [26]. Specific inhibitors of actin, like cytochalasin D and jasplakinolide, were shown to inhibit the encystation of *E. invadens* [27,28]. To find the involvement of actin in cyst chitin wall formation, actin was localized using rhodamine phalloidin in encysting cells.

**Figure 7:**
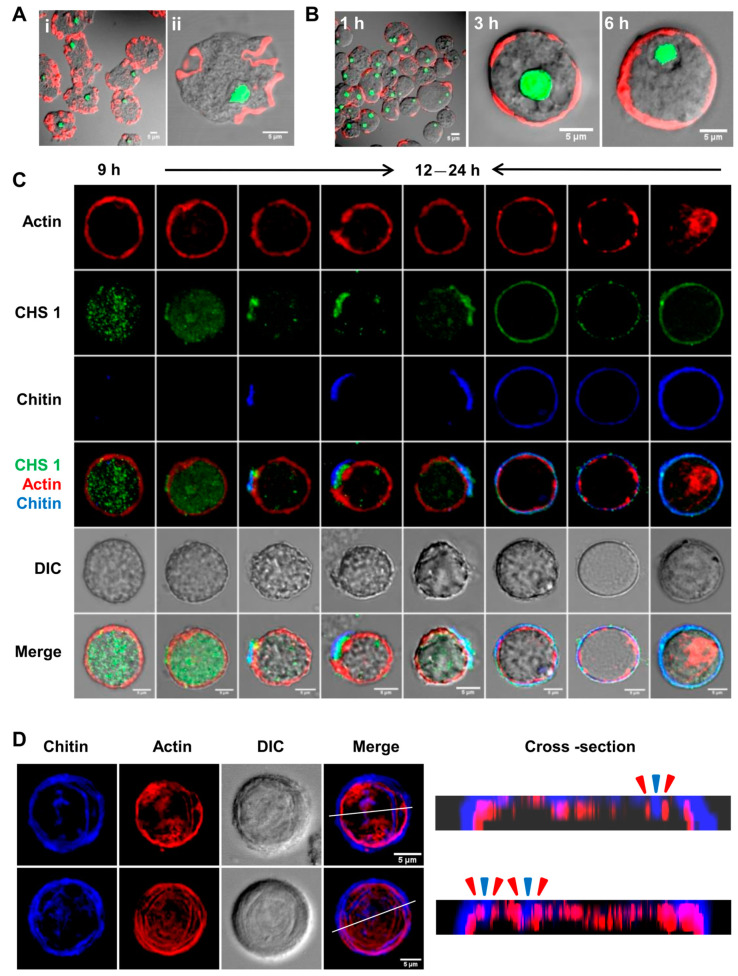
Reorganization of actin cytoskeleton during encystation. (**A**) In trophozoites, actin was mainly found in the phagocytic and pinocytic invaginations (i,ii). Red and green colors represent actin and nucleus, respectively. (**B**) During initiation of encystation, all the actin polymerized and relocalized at multiple points in the cell’s cortical region after 1 h, forming an incomplete ring after 3 h and turning into a fully formed cortical actin ring by the end of the 6 h period. Red and green colors represent actin and nucleus, respectively. (**C**) Chitin synthase vesicles were found in the cytoplasm by the 9th hour. Between 12 and 24 h, chitin synthase was observed to accumulate on the cortical actin, and, at these sites, chitin synthesis started. The chitin wall then grew as more chitin syntheses add chitin to the growing wall. Chitin synthase remained in the wall after it was completed, but the cortical actin was observed to disassemble afterward. Green, red, and blue colors represent chitin synthase 1, actin, and chitin, respectively. (**D**) Linear chitin fibrils were deposited between and above the actin ridges, which can be seen from the cross-section of the cyst surface. In cross-sections, the blue and red arrowhead represent the chitin and actin. Blue and red colors represent chitin and actin, respectively. Scale bar (white line): 5 µm.

**Figure 8:**
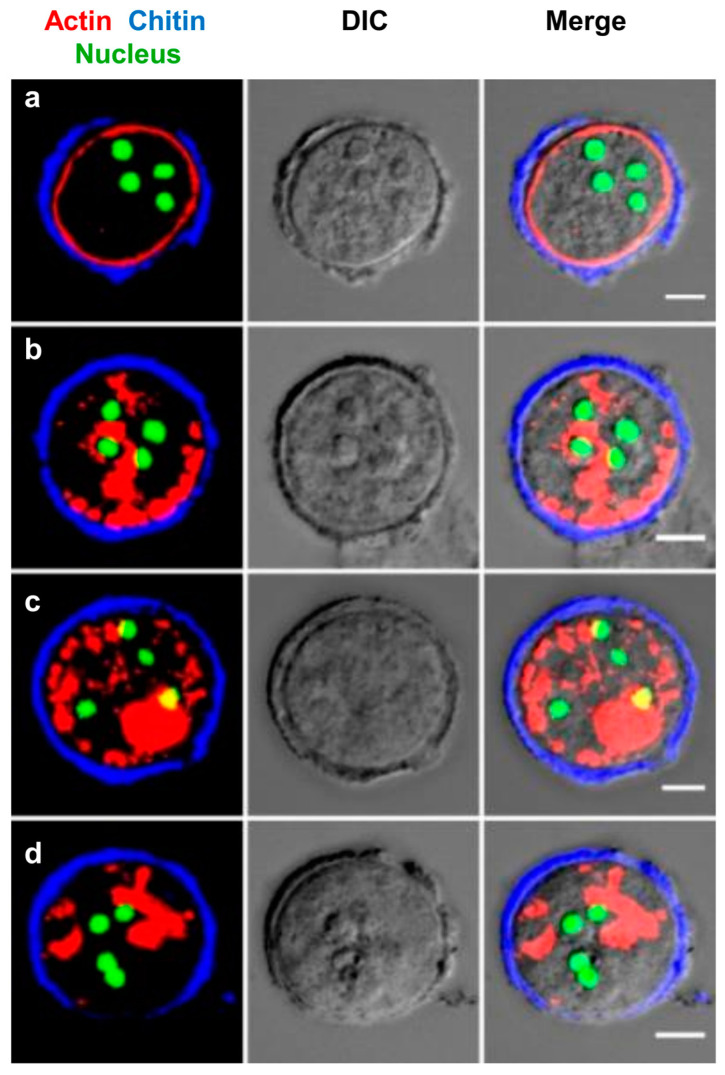
Localization of cortical actin after wall formation. Actin is present as a cortical ring in cysts with incomplete chitin cell walls (**a**). But, after the completion of the wall in 48–72 h mature cysts, cortical actin rings change to form actin aggregates in the cytoplasm (**b**–**d**). Red, blue, and green colors represent actin, chitin, and nucleus, respectively. Scale bar (white line): 5 µm.

In trophozoites, actin was mainly localized in the phagocytic/pinocytic invaginations found all over the surface (Figure 7A—i,ii). But, during the initiation of encystation, all the actin was observed to polymerize in the cortical region (Figure 7B). After 1 h of the encystation process, polymerized actin was detected at various locations within the cell’s cortical region. After 3 h, an incomplete cortical actin ring was observed. This ring formation continued to progress, and, by the end of the 6 h encystation period, a fully formed cortical actin ring was evident. Uniform actin polymerization at the cortical region is reported to cause isotropic contraction during mitotic cell rounding [48], and the same may also be responsible for the round morphology of cysts. Further studies indicated that chitin synthase was present in the vesicles in the cytoplasm by the 9th hour of encystation, and, between 12 and 24 h, they started to accumulate on the cortical actin that eventually guides chitin synthesis and chitin wall formation over this cortical actin (Figure 7C). As previously shown (Figure 4A), more chitin synthase reached the cyst surface, depositing chitin and extending the wall. As described earlier, chitin is deposited in a patterned manner on the cyst surface, and this pattern was also reflected in the cortical actin cytoskeleton during the early hours (Figure 7D). A cross-section of the cyst surface obtained from confocal z-stacks showed that chitin deposition took place between and above F-actin ridges (Figure 7D). Thus, the actin cytoskeleton may have a role in guiding the location for chitin fibril deposition on the cyst surface, similar to the cytoskeleton-guided cellulose deposition in plant cell walls [49]. The cortical actin cytoskeleton may also be involved in the transport and secretion of cyst wall components, as observed during the encystation of *Giardia intestinalis* [50]. Changes in cortical actin localization were only observed after the completion of the wall. When mature cysts obtained from 48 h encystation culture were stained with rhodamine phalloidin (Figure 8), actin remained in the cell cortex in a few cysts (Figure 8a), but, in most cases, it formed aggregates of different sizes in the cytoplasm (Figure 8b–d). Most of the encysting cells demonstrated that cortical actin becomes disassembled after wall formation is completed, indicating a temporal relationship between cortical actin and chitin deposition.

### 3.5. 2,3-Butanedione Monoxime Treatment Inhibited Cortical Actin Formation and Produced Wall-Less Cysts and Cysts with Defective Walls

To understand the role of cortical actin in *Entamoeba* cyst wall deposition, actin polymerization was disrupted by 2,3-Butanedione monoxime (BDM), as it has been used to study the synthesis and organization of yeast chitin cell wall [51,52]. It was observed to disorganize the polar distribution of actin patches in *Schizosaccharomyces pombe*, resulting in an uneven cell wall [51]. BDM inhibits actomyosin contractility by blocking the ATPase activity of myosin II [53] and is also found to specifically inhibit the myosin-dependent functions in *E. histolytica* [54].

**Figure 9:**
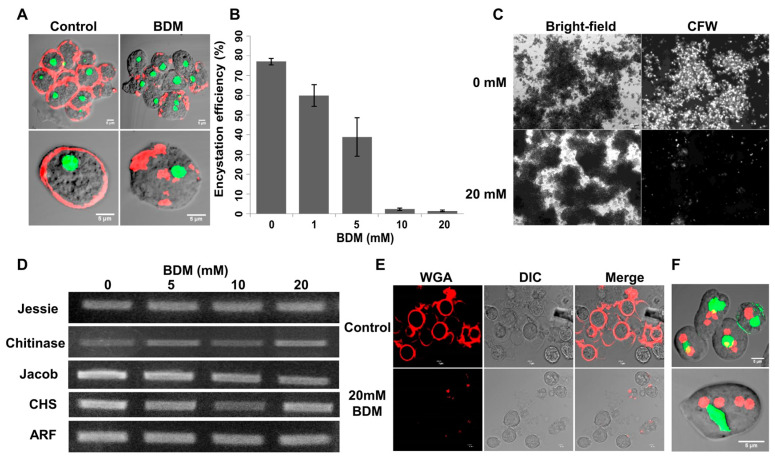
Effects of 2, 3-Butanedione monoxime on encystation. (**A**) Cortical actin was found in encysting cells by 2 h, but, in BDM-treated cells, actin was found as cytoplasmic aggregates. Red and green colors represent actin and nucleus, respectively. Scale bar (white line): 5 µm. (**B**) BDM dose-dependently reduced encystation efficiency. (**C**) Cysts were only formed inside the cell aggregates during encystation. In the treated culture, aggregates still formed, but fewer cysts were found in them. Scale bar: 100 µm. (**D**) Semi-quantitative RT-PCR with mRNA isolated from 24th-hour encysting cells showed that all cyst wall genes are expressed in BDM-treated encystation culture. ARF gene was used as an internal control. (**E**) Control and BDM-treated cysts were obtained from a 48th-hour encystation culture after staining with WGA (red). BDM treatment caused aberrant chitin deposition. The red color represents chitin. (**F**) Nuclear staining with PI (red) and indirect staining of the chromatoid body with FITC (green) showed a large number of tetranucleated “wall less cysts” in culture. Red and green colors represent the nucleus and chromatoid body, respectively. Scale bar (white line): 5 µm. (Data are shown as mean ± SD for a minimum of 3 independent experiments).

At a 20 mM concentration, BDM inhibited the rearrangement of cellular actin into the cell cortex, as shown by rhodamine phalloidin staining. In BDM-treated cells, actin was observed to form an aggregate in the cytoplasm (Figure 9A). When added to the encystation culture, BDM dose-dependently inhibited encystation, with 20 mM of BDM reducing the encystation efficiency to <5% (Figure 9B). In the encystation culture, cysts are only formed inside the galactose-ligand-mediated cell aggregates formed during encystation, and the cell signaling required for encystation takes place in these aggregates [55]. The addition of BDM did not inhibit cell aggregation, but only a few chitin-walled cysts were found in these aggregates, as shown by CFW staining (Figure 9C). However, in BDM-treated cells, no change in the expression level of cyst wall proteins was observed by RT-PCR (Figure 9D), and WGA staining showed many cells with disordered and patchy localization of chitin on their surface (Figure 9E). Further staining of BDM-treated cells for the nucleus and chromatoid body revealed that most cells contain the chromatoid body and four nuclei, indicating the cells entered the encystation process and formed cysts without a cell wall (Figure 9F). The presence of many tetranucleate cells with the chromatoid body indicated that BDM treatment affects cyst wall formation without preventing the other events of cyst maturation. The presence of cysts with aberrant walls and “wall-less cysts” in BDM-treated cultures showed that the cortical actin cytoskeleton is necessary for proper chitin deposition. These observations also showed that chitin wall deposition and other developmental events took place independently of each other.

### 3.6. Cytochalasin D Treatment Inhibited Cortical Actin Formation and Produced Wall-Less Cysts and Cysts with Defective Walls

In order to enhance comprehension of the function of cortical actin in the deposition of the *Entamoeba* cyst wall, as demonstrated by BDM, cytochalasin D, a cell-permeable fungal toxin that inhibits actin polymerization, was also employed to disrupt actin polymerization. It inhibited functions dependent on microfilaments in *E. histolytica* [56]. Additionally, it was employed to investigate *E. invadens*’ proliferation, encystation, and multinucleation. A dose-dependent inhibition of proliferation and encystation was observed in response to cytochalasin D [27,57].

**Figure 10:**
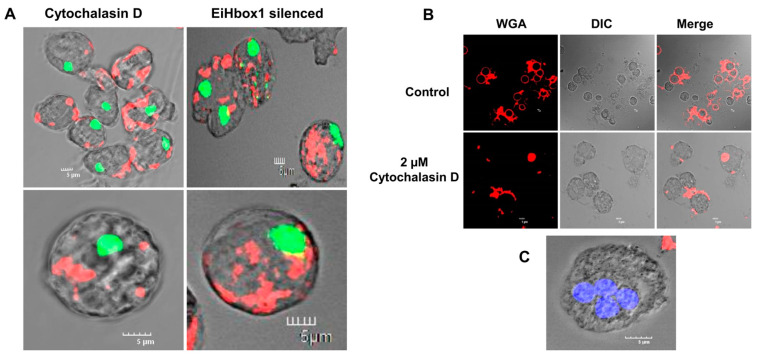
Effects of cytochalasin D and EiHbox1 on F-actin and encystation. (**A**) In cytochalasin D-treated and EiHbox1-silenced cells, F-actin was found as cytoplasmic aggregates. Red and green colors represent actin and nucleus, respectively. Scale bar (white line): 5 µm. (**B**) Control and cytochalasin D-treated cysts were obtained from a 48th-hour encystation culture after staining with WGA (red). Cytochalasin D treatment caused aberrant chitin deposition. Red color represents chitin. (**C**) Nuclear staining with DAPI (blue) showed tetranucleated “wall-less cysts” in the culture. Blue color represents nucleus. Scale bar (white line): 5 µm.

In addition, 2 µM cytochalasin D inhibited the rearrangement of cellular actin into the cell cortex, as revealed by rhodamine phalloidin staining. The formation of actin aggregate in the cytoplasm was observed in cells treated with cytochalasin D (Figure 10A). The encystation process was inhibited when cytochalasin D was introduced into the culture. Upon WGA staining, numerous cells exhibited patchy and disordered chitin localization on their surfaces (Figure 10B). In addition, the nucleus staining of cytochalasin D-treated cells revealed that the majority of cells contained all four nuclei, indicating that the cells had entered the encystation process and developed into cysts without a cell wall (Figure 10C). Numerous tetranucleate cells were observed, suggesting that cytochalasin D treatment had an impact on cyst wall formation but did not impede the progression of other cyst maturation processes, similar to BDM-treated cells. Cysts with abnormal walls and “wall-less cysts” were observed in cultures treated with cytochalasin D, supporting observations of BDM treatment that the cortical actin cytoskeleton is an essential component for the appropriate deposition of chitin. Furthermore, these observations also demonstrated that chitin wall deposition and other developmental processes autonomously occur.

### 3.7. Silencing of TALE Homeobox Transcription Factor EiHbox1 Inhibits Cortical Actin Formation

The EiHbox1 gene [31], which encodes a TALE homeobox transcription factor in the protozoan parasite *E. invadens*, had significant effects on the process of cyst formation. EiHbox1 is known to regulate encystation by controlling the expression of specific genes related to this process. When EiHbox1 was silenced (98% knockdown efficiency), the efficiency of encystation in the parasites was reduced, and the morphology of the resulting cysts was also altered [31]. Further, promoter analysis shows that along with encystation-related genes, EiHbox1 binding sites are also present in cytoskeletal-related genes. The transcriptomics data of the EiHbox1-silenced cell line showed the downregulation of some important cytoskeletal-related genes, suggesting a regulatory role for EiHbox1 in cytoskeletal dynamics during encystation [31].

To further confirm the role of the actin cytoskeleton in the formation of proper chitin walls in *E. invadens*, F-actin was stained with rhodamine phalloidin in encysting EiHbox1-silenced cells. The silencing of EiHbox1 had an impact on the organization of cellular actin. In EiHbox1-silenced parasites, F-actin was not arranged in the cortical region. Instead, actin was found to aggregate in the cytoplasm, resembling the pattern seen in cells treated with compounds like BDM and cytochalasin D (Figure 10A). This observation highlights the crucial role of EiHbox1 in orchestrating the proper arrangement of cortical actin during the formation of *Entamoeba* cyst walls. Moreover, it emphasizes the significance of cortical actin in the process of *Entamoeba* cyst wall formation.

### 3.8. Inhibition of Cortical Actin Formation Affected Both Localization and Activity of Chitin Synthase

**Figure 11:**
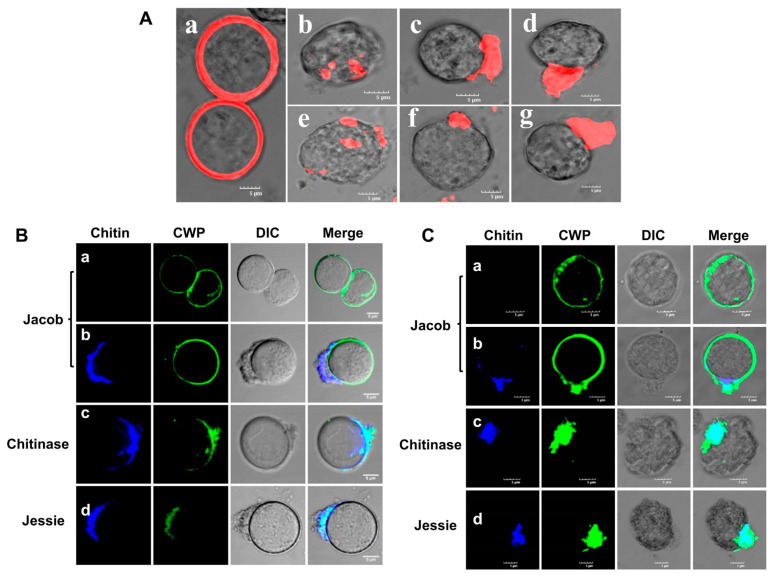
Localization of chitin and cyst wall proteins (CWP) in BDM- and cytochalasin D-treated cysts. (**A**) Chitin wall is of uniform thickness in control cysts (a). BDM treatment caused aberrant cyst wall formation and uneven chitin deposition (b) and clustering of chitin fibrils to one end of the cell (c,d). Similarly, cytochalasin D treatment also caused aberrant cyst wall formation, uneven chitin deposition (e), and clustering of chitin fibrils to one end of the cell (f,g). Red color represent chitin. (**B**) Confocal microscopy showed that in BDM-treated cysts, no change in the secretion or localization of Jacob occurred. Jacob was found on the surface of cysts with no chitin wall or with aberrant chitin deposition. Chitinase and Jessie were always co-localized with chitin, since they were secreted outside and contained chitin-binding domains. (**C**). Confocal microscopy showing no further change in the secretion or localization of Jacob in cytochalasin D-treated cysts. Jacob was found on the surface of cysts with no chitin wall or with aberrant chitin deposition. Chitinase and Jessie were always co-localized with chitin, since it was secreted outside and contained chitin-binding domains. Blue and green colors represent chitin and cyst wall protein, respectively. Scale bar (white line): 5 µm.

BDM- and cytochalasin D-treated encystation culture contained both “wall-less cysts” and cysts with abnormal walls. Instead of forming a uniform chitin wall (Figure 11A—a), in BDM-treated cells, chitin was deposited in an irregular fashion (Figure 11A—b), but, in most cases, it was found to be accumulated on one side (Figure 11A—c,d). Analogous to cells treated with BDM, cytochalasin D-treated cells also exhibited an irregular deposition pattern of chitin (Figure 11A—e), with the majority of cases revealing its accumulation on a single side (Figure 11A—f,g). To find how the cortical actin disruption affects cyst wall formation, the cyst wall proteins were localized (Figure 11B,C). The immunostaining of Jacob in BDM- and cytochalasin D-treated cells showed no change in its localization; it was present on the cyst surface in both ‘‘wall-less cysts’’ (Figure 11B—a,C—a) and cysts with aberrant walls (Figure 11B—b,C—b), just like in normal cysts. Jacob secretion, or its binding to the Gal/GalNAc lectin and, thus, the ‘‘foundation’’ phase of encystation, was not affected by BDM or cytochalasin D. The immunolocalization of chitinase (Figure 11B—c,C—c) and Jessie (Figure 11B—d,C—d) in BDM- and cytochalasin D-treated cells with their respective antibodies showed that these proteins co-localized with aberrant chitin walls like normal chitin walls. This could be because these proteins were secreted to the extracellular space and became attached to chitin fibrils through their chitin-binding domains. Thus, the secretions of cell wall components were not affected, only the deposition of chitin was, which indicated a link between cortical actin and chitin synthase localization. In temperature-sensitive actin mutants of yeasts, the dysfunction of actin led to the formation of an aberrant wall by delocalizing the chitin synthase [58,59]. Actin inhibitors caused the mislocalization of chitin synthase in *Neurospora crassa*, indicating the role of actin in the traffic and localization of chitin synthase [60].

**Figure 12:**
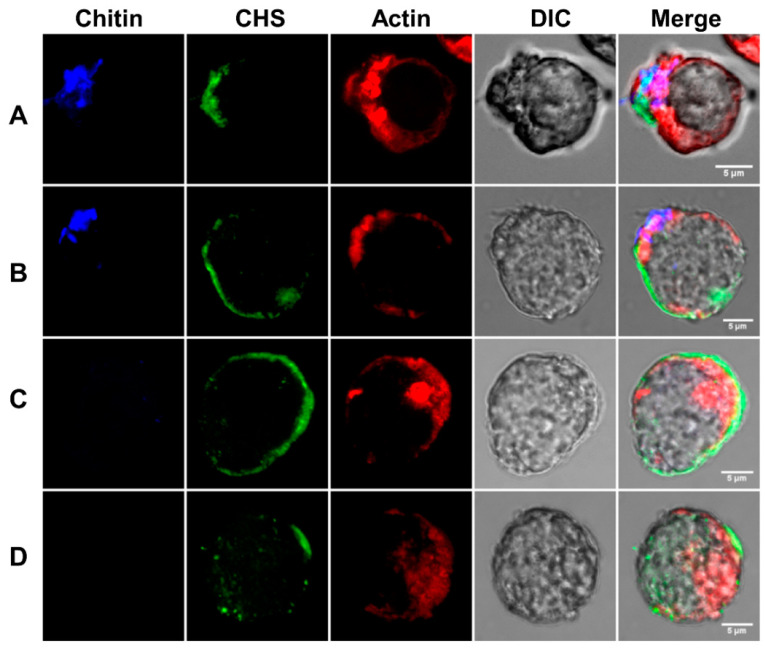
Immunolocalization of chitin synthase in BDM-treated cells. Actin remained delocalized in BDM-treated cells. Uneven chitin deposition was observed in some cells (**A**,**B**); others did not form chitin, even though chitin synthase was observed on the cell surface (**C**,**D**). Blue, green, and red colors represent the chitin wall, chitin synthase 1, and actin, respectively. Scale bar (white line): 5 µm.

The immunolocalization of chitin synthase (EiCHS1) in BDM-treated cells obtained from a 72 h encystation culture showed that it was present on the surface of most cells, both cysts with aberrant chitin walls (Figure 12A,B) and wall-less cysts (Figure 12C,D). Thus, the effects of BDM on cell wall deposition resulted from its inhibition of chitin synthase localization and/or its activity, indicating a relationship between the cortical actin cytoskeleton and chitin synthase 1. In *Aspergillus nidulans*, chitin synthase was concentrated at sites of chitin synthesis like hyphal tips and septation sites. This localization required interaction between the actin cytoskeleton and myosin motor-like domain (MMD) of chitin synthase, and the MMD–actin interaction was necessary for both the localization and function of chitin synthase [61]. The function of the cortical actin cytoskeleton may be to anchor chitin synthases and act as a scaffold to maintain the structure. Since EiCHS 1 did not contain any of the MMD, further studies are required to find the exact nature of the interaction between the actin cytoskeleton and chitin synthase, if there is any.

### 3.9. Modified Cyst Wall Synthesis Pathways during Entamoeba Encystation

**Figure 13:**
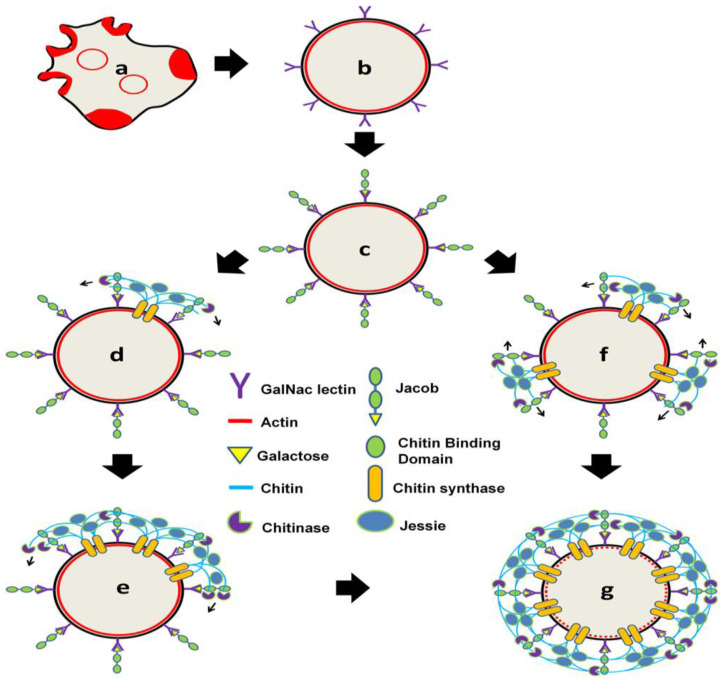
Model of chitin wall formation. F-actin present in the amoeba cells (**a**) is reorganized into cortical regions within 1 h of encystation (**b**). Jacob lectin is secreted (**b**) and binds to Gal/GalNAc lectins (**c**). Chitin synthase produces chitin fibrils at one point (**d**) or multiple points (**f**). Secreted chitinases and Jessie binds to this framework. Cyst wall grows from these points (**e**) and, finally, covers the whole surface (**g**). The major pathway is a-b-c-d-e-g, and the minor pathway is a-b-c-f-g.

The modified Wattle and Daub model of cyst wall synthesis is shown in Figure 13. In the majority of *Entamoeba* trophozoites, polymerized actin was distributed across the cell surface within phagocytic/pinocytic invaginations (Figure 13a). As the cells initiated the process of encystation, they underwent morphological transformations, leading to a comprehensive reorganization of the actin cytoskeleton. This reorganization resulted in the formation of a cortical actin ring positioned beneath the plasma membrane (Figure 13b). During encystation, the cells secreted the Jacob lectin, which bound to the Gal/GalNac lectins constitutively expressed on the cell surface. This interaction covered the cell surface and established the foundation for the synthesis of the cyst wall (Figure 13c). Subsequently, chitin synthase was transported to the plasma membrane, where it clustered on the cortical actin, either at a singular point or at multiple points where chitin synthesis commenced. Chitin synthase generated chitin fibrils, to which the Jacob lectin was bound through its chitin-binding domain (CBD), forming the initial framework of the cyst wall (Figure 13d,f). Also, other proteins like chitinase and the Jessie lectin, secreted in the early stages, bound to these partially formed chitin walls due to their chitin-binding domains. These proteins remained embedded in the wall even after its synthesis. Chitinase played a role in trimming chitin fibrils, while the Jessie lectin contributed to making the wall impermeable. The cyst wall continued to grow from these singular or multiple points (Figure 13e,f), ultimately covering the entire cell surface (Figure 13g). Upon the completion of the cyst wall, the cortical actin underwent a transformation into cytoplasmic aggregates. The predominant route for the synthesis of the cell wall in the majority of *Entamoeba* trophozoites during encystation involves a sequential progression through the steps denoted as a-b-c-d-e-g. In this pathway, the cortical actin rearranges, and the cell wall synthesis initiates at a single point, eventually extending and covering the entire cell surface. Conversely, a less common pathway, represented by a-b-c-f-g, is observed in a minority of cells. In this alternative route, the initiation of cell wall synthesis occurs at multiple points on the cortical actin.

In conclusion, this study explored cyst wall formation in more detail, in relation to chitin deposition on the cyst wall and chromatoid body formation, and the role of chitin synthase was reported for the first time. Various aspects of the actin scaffold, with respect to cyst wall formation, were also demonstrated at the cellular level. The Wattle and Daub model of *Entamoeba* cyst wall biosynthesis was revised, and the modified model is explained in Figure 13.

## Figures and Tables

**Table 1:** Primers for RT-PCR.

Gene/ID	Primers (5′-3′)
ADP Ribosylation Factor EIN_268250	EiARF F: 5′ CCATCATCTTTGTAGTTGATTCCA 3′
	EiARF R: 5′ TCACTTGAGAGTGTCAGCAAGC 3′
Jacob EIN_230100	EiJac F: 5′ TACTGAAACGTCCAAAGAAGAGTC 3′
	EiJac R: 5′ TTAATTCTTCTTTGCCCAGGTT 3′
Chitinase EIN_096870	Ei Chi3 F: 5′ GATGGCAAACATCGAAATCG 3′
	Ei Chi3 R: 5′ TTAGTTTTTCAACTCATCG 3′
Jessie EIN_066080	EiJes3a F: 5′ CTTGCTGTGCCTTGCTTTAA 3′
	Ei Jes3a R: 5′ CCCCAAATACTTCCC TGGT 3′
Chitin Synthase 1 EIN_066020	Ei CHS1 F: 5′ TAATGATATTGGGTGTCGTTCA3′
	Ei CHS1 R: 5′ TATACACCACCACGAGTCCC 3′

## Data Availability

Data are contained within the article.
